# The Role of the Axial Substituent in Subphthalocyanine Acceptors for Bulk‐Heterojunction Solar Cells

**DOI:** 10.1002/anie.201608644

**Published:** 2016-11-28

**Authors:** Chunhui Duan, Germán Zango, Miguel García Iglesias, Fallon J. M. Colberts, Martijn M. Wienk, M. Victoria Martínez‐Díaz, René A. J. Janssen, Tomás Torres

**Affiliations:** ^1^ Molecular Materials and Nanosystems Institute for Complex Molecular Systems Eindhoven University of Technology P. O. Box 513 5600 MB Eindhoven The Netherlands; ^2^ Department of Organic Chemistry Universidad Autónoma de Madrid c/Francisco TomásyValiente 7 Cantoblanco 28049 Madrid Spain; ^3^ IMDEA-Nanociencia c/Faraday 9, Campus de Cantoblanco 28049 Madrid Spain; ^4^ Dutch Institute for Fundamental Energy Research De Zaale 20 5612 AJ Eindhoven The Netherlands

**Keywords:** bulk heterojunctions, electron acceptors, solar cells, subphthalocyanines, substitution effects

## Abstract

Four hexachlorosubphthalocyanines SubPcCl_6_‐X bearing different axial substituents (X) have been synthesized for use as novel electron acceptors in solution‐processed bulk‐heterojunction organic solar cells. Subphthalocyanines are aromatic chromophoric molecules with cone‐shaped structure, good solution processability, intense optical absorption in the visible spectral region, appropriate electron mobilities, and tunable energy levels. Solar cells with subphthalocyanines as the electron acceptor and PTB7‐Th as the electron donor exhibit a power conversion efficiency up to 4 % and an external quantum efficiency approaching 60 % due to significant contributions from both the electron donor and the electron acceptor to the photocurrent, indicating a promising prospect of non‐fullerene acceptors based on subphthalocyanines and structurally related systems.

Solution‐processed bulk‐heterojunction (BHJ) organic solar cells (OSCs) are a promising renewable‐energy technology towards future efficient, large‐area, flexible photovoltaic modules.^1^ The main component of an OSC is its BHJ‐active layer, consisting of an electron‐donor and an electron‐acceptor phase separated into a bicontinuous interpenetrating network morphology.^2^ Power conversion efficiencies (PCEs) exceeding 11 % have been achieved recently.^3^ While numerous electron donors, including semiconducting polymers and small molecules, have been assessed,^4^ electron‐acceptor components are still dominated by fullerene derivatives because of their high electron mobility, ideal frontier orbital energy levels, and isotropic charge‐transport properties.^5^ However, fullerene derivatives have intrinsic shortcomings, such as high cost of synthesis, low absorption coefficients in the visible spectral region, limited variability in the energy levels, and morphological instability in the blended films.^6^ The development of new electron acceptors which overcome the drawbacks associated with fullerene‐based acceptors is thus vital for further advancing OSCs.^7^ Encouragingly, several studies have reported BHJ solar cells with PCEs >8 % based on non‐fullerene acceptors.^8^

Subphthalocyanines (SubPcs) are aromatic chromophoric molecules with a boron atom at their inner cavity, intense optical absorption in the 460–580 nm spectral region,^9^ and relatively high electron mobilities.^10^ Traditionally, they have been used as electron donors in vacuum‐deposited planar‐heterojunction solar cells.9a However, the electronic properties of SubPcs can be easily adjusted by introducing axial and/or peripheral substituents.9a Hence, by rational molecular design, that is, introducing peripheral electron‐withdrawing groups, SubPcs have been transformed into electron‐acceptor molecules.^11^ For example, non‐fullerene vacuum‐evaporated solar cells containing SubPc molecules achieved a PCE of 8.4 %.^12^ The cone‐shaped structure of SubPcs prevents excessive aggregation in solution and in the solid state, providing good solution processability even without the assistance of electrically insulating alkyl chains. Nevertheless, only very recently, SubPc molecules have been demonstrated as electron acceptors in solution‐processed BHJ solar cells.^13^

Herein, we report on four hexachlorosubphthalocyanines (SubPcCl_6_‐X) bearing different axial substituents, that is, chlorine and differently substituted phenoxy groups, as electron acceptors in BHJ solar cells. SubPcCl_6_‐Cl has demonstrated great potential as n‐type material in planar‐heterojunction OSCs.^11^ Modification of the axial substituent results in a slight variation of the electron‐accepting character of the molecule and can greatly affect its aggregation and crystallization behavior. A maximum PCE of 4.0 % has been achieved for SubPcCl_6_‐Cl, which is the highest value for solution‐processed solar cells based on SubPc derivatives. This work paves the way to a new class of non‐fullerene acceptors with tunable optoelectronic properties and microstructures for efficient BHJ OSCs.

Four axially substituted SubPcCl_6_‐X derivatives, shown in Figure 1, were used in this study. SubPcCl_6_‐Cl was prepared according to a literature procedure.11d Reaction with selected phenol derivatives with electron‐donating or ‐withdrawing substituents provided SubPcCl_6_‐OC_6_H_2_(OMe)_3_, SubPcCl_6_‐OC_6_H_4_^*t*^Bu, and SubPcCl_6_‐OC_6_F_5_ in good yields (see the Supporting Information (SI) for synthetic procedures).

**Figure 1:**
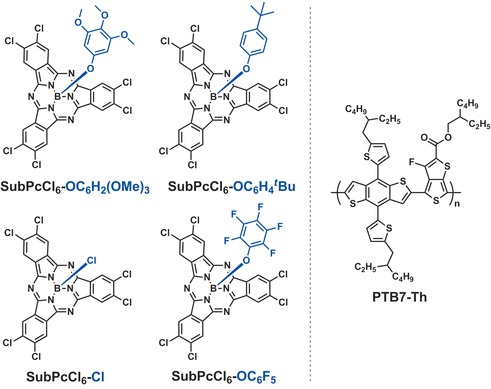
Chemical structures of the electron‐acceptor SubPc derivatives and the donor polymer PTB7‐Th used in this work.

All four SubPc compounds feature almost identical absorption spectra in toluene, signifying a small influence of the axial substituents. The Q‐band peaks are found at wavelengths around 570 nm with absorption coefficients of 4.0×10^4^–4.5×10^4^ m ^−1^ cm^−1^ (Figure S2, Table 1). In films, the absorption spectra of SubPcCl_6_‐X (Figure 2 a) show a bathochromic shift of about 10 nm compared to the spectra in solution (Table 1). Remarkably, the SubPcCl_6_‐Cl absorption spectrum in film displays a broader Q band with an additional intense maximum at 558 nm, which could be attributed to the formation of head‐to‐tail columnar stacks (H‐type‐like aggregates) in the solid state, favored by the presence of the small chlorine atom in the axial position. Axial substitution with bulky phenoxy groups in the other three SubPcCl_6_‐X derivatives precludes this behavior. The fluorescence quantum yield *ϕ*_F_ of SubPcCl_6_‐Cl, SubPcCl_6_‐OC_6_H_4_^*t*^Bu and SubPcCl_6_‐OC_6_F_5_ in toluene is around 0.35–0.64, but it is only 0.003 for SubPcCl_6_‐OC_6_H_2_(OMe)_3_. The strong quenching in the latter is attributed to an intramolecular photoinduced electron‐transfer process from the electron‐rich axial phenoxy substituent to the SubPc acceptor.

**Figure 2:**
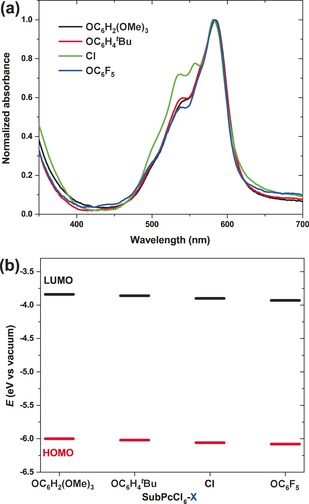
SubPcCl_6_‐X: a) optical absorption spectra of their films and b) their energy levels.

**Table 1:** Optical properties and energy levels of the boron subphthalocyanine derivatives SubPcCl_6_‐X.

X	*λ*_max_ [nm]		*E*_g_^opt^ [eV]	HOMO [eV]	LUMO [eV]	*ϕ*_F_
	solution	film				
C_6_H_2_(OMe)_3_	572	584	2.16	−6.00	−3.84	0.003
OC_6_H_4_^*t*^Bu	571	584	2.16	−6.02	−3.86	0.351
Cl	574	582	2.16	−6.06	−3.90	0.643
OC_6_F_5_	572	582	2.15	−6.08	−3.93	0.429

Electrochemical properties of the four SubPcCl_6_‐X derivatives were investigated by cyclic voltammetry (CV) and square‐wave voltammetry (SWV) in tetrahydrofuran (Figure S4 (SI), Figure 2 b, and Table 1). The LUMO and HOMO energy levels were estimated from the reduction potentials obtained by CV measurements and optical‐band‐gap (*E*_g_^opt^) values. While peripheral electron‐withdrawing chlorine substituents govern SubPcCl_6_‐X electron affinities, axial substitution allows fine‐tuning of the LUMO and HOMO levels in a 0.1 eV range, resulting in LUMO and HOMO energies spanning from −3.84 and −6.00 eV, respectively, for SubPcCl_6_‐OC_6_H_2_(OMe)_3_ to −3.93 and −6.08 eV, respectively, for SubPcCl_6_‐OC_6_F_5_ (Table 1).

The photovoltaic properties of the SubPc molecules were evaluated in solar cells with an ITO/ZnO/PTB7‐Th:SubPcCl_6_‐X/MoO_*x*_/Ag device architecture under simulated AM1.5G illumination (100 mW cm^−2^). The current density–voltage (*J*–*V*) characteristics and external quantum efficiency (EQE) spectra of the optimized devices are shown in Figure 3 and summarized in Table 2. To accurately determine the PCEs of the solar cells, the short‐circuit current density (*J*_sc_) was obtained by integrating the EQE with the AM1.5G spectrum. Results from different fabricating conditions and device statistics can be found in Table S1 and S2. Solar cells with a structure that uses either ITO/PEDOT:PSS or ITO/MoO_x_ as bottom contacts all produced very poor results, possibly caused by an unfavorable vertical phase segregation (Figure S5). The highest PCE of 4.0 % was found for SubPcCl_6_‐Cl, which offered a *J*_sc_ of 10.7 mA cm^−2^, an open‐circuit voltage (*V*_oc_) of 0.77 V, and a fill factor (FF) of 0.48. In each case both the polymer donor and the SubPc acceptor contribute substantially to the photocurrent (Figure 3 b). The EQE maxima located at about 710 nm originate from PTB7‐Th, while the peaks at about 570 nm result from the SubPc molecules. The lack of light absorption of PTB7‐Th and SubPcCl_6_‐X between 350 and 450 nm results in a rather uncommon shape in the EQE spectra, with a valley in this region. At higher wavelengths, an impressive EQE maximum of 0.58 was achieved for SubPcCl_6_‐Cl, while the other SubPc molecules produced significantly lower EQE and *J*_sc_ values. As a result, these devices all afforded PCEs below 2.0 %. Notably, except for SubPcCl_6_‐OC_6_F_5_, the SubPc solar cells exhibit a similar *V*_oc_ value (about 0.8 V) as PTB7‐Th:fullerene devices. Table 2 also shows that a common limitation of these solar cells is their low FF (<0.5), which is considerably lower than that of the state‐of‐the‐art fullerene‐based devices. Understanding the origin of the low FF of these devices is crucial to further develop SubPc‐based acceptors.

**Figure 3:**
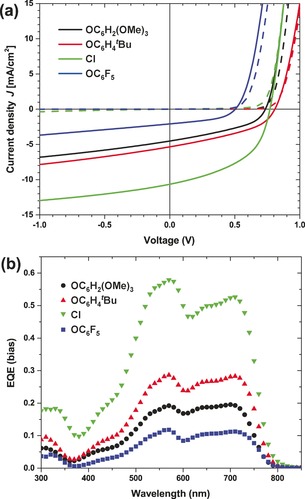
a) *J*–*V* curves of the PTB7‐Th:SubPcCl_6_‐X solar cells in dark (dashed lines) and under illumination (solid lines); b) corresponding EQE spectra.

**Table 2:** Solar‐cell characteristics and electron mobilities of PTB7‐Th:SubPcCl_6_‐X devices.

X	*J*_sc_^[a]^ [mA cm^−2^]	*V*_oc_ [V]	FF	PCE [%]	EQE_max_	*μ*_e_^[b]^ [cm^2^ V^−1^ s^−1^]
C_6_H_2_(OMe)_3_	3.6	0.74	0.40	1.1	0.20	1.5×10^−6^
OC_6_H_4_^*t*^Bu	5.3	0.81	0.41	1.8	0.29	5.1×10^−6^
Cl	10.7	0.77	0.48	4.0	0.58	8.3×10^−6^
OC_6_F_5_	2.1	0.50	0.45	0.5	0.12	1.6×10^−6^

[a] Determined by integrating the EQE spectrum with the AM1.5G spectrum. [b] Electron mobilities were measured in electron‐only devices.

Hence, the charge‐carrier transport properties and the bimolecular charge recombination of the PTB7‐Th:SubPcCl_6_‐X blend films were investigated. Electron mobilities (*μ*_e_) (Table 2) were estimated from electron‐only PTB7‐Th:SubPcCl_6_‐X devices by fitting the *J*–*V* data (Figure S6) to a space‐charge‐limited current model, resulting in *μ*_e_≈10^−6^ cm^2^ V^−1^ s^−1^ for all four acceptors. Keeping in mind that PTB7‐Th:fullerene blends exhibit a *μ*_e_ of 10^−3^–10^−2^ cm^2^ V^−1^ s^−1^,^14^ the considerably lower *μ*_e_ values of the PTB7‐Th:SubPcCl_6_‐X films are likely causing the low FF.

As a result of the low *μ*_e_ values, PTB7‐Th:SubPcCl_6_‐X solar cells exhibit substantial bimolecular charge‐recombination losses. These are evidenced by the large difference between the EQE measured with light bias (EQE_bias_) and without light bias (EQE_nobias_) (Figure S7). The average values for *ρ*=EQE_bias_/EQE_nobias_ (Figure 4) can be used to estimate the bimolecular recombination efficiency via *η*_BR_=1−*ρ*.^15^ Hence a low *ρ* value indicates considerable bimolecular recombination. For state‐of‐the‐art polymer:fullerene solar cells *ρ* approaches unity. The low *ρ* values of these PTB7‐Th:SubPcCl_6_‐X devices evidence severe bimolecular recombination losses, even at short circuit. The highest *ρ* value is found for PTB7‐Th:SubPcCl_6_‐Cl, which is consistent with the higher *μ*_e_ value, FF, and PCE of the SubPcCl_6_‐Cl solar cell. We speculate that the higher performance originates from the H‐type‐like aggregates of SubPcCl_6_‐Cl in the solid state, which are favorable for charge transport.

**Figure 4:**
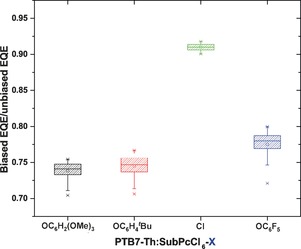
Average EQE_bias_/EQE_nobias_ values of PTB7‐Th:SubPcCl_6_‐X solar cells.

Next to bimolecular recombination, also geminate recombination can result in a low FF. The current density of illuminated solar cells shows a substantial increase under reverse bias. This can be a consequence of the enhanced internal electric field promoting charge separation from the donor–acceptor interface.

The morphology of PTB7‐Th:SubPcCl_6_‐X blend films was investigated by transmission electron microscopy (TEM). The blends based on SubPcCl_6_‐OC_6_H_4_^*t*^Bu and SubPcCl_6_‐Cl show homogeneous films without noteworthy phase separation (Figure 5 b and c). In intimately mixed blends, charge separation is prevented because of the lack of pure domains. High domain purity is beneficial for dissociating photogenerated charges from the donor–acceptor interface, while low domain purity often causes serious geminate recombination.^16^ In the films based on SubPcCl_6_‐OC_6_H_2_(OMe)_3_ and SubPcCl_6_‐OC_6_F_5_, there is a slightly increased contrast between the light and dark regions, indicating a more distinct phase separation, but these films lack long‐enough fibrillary structure (Figure 5 a and d). Charge‐carrier transport in such films may thus be impeded because of a poor interconnectivity between neighboring domains. Combined, the low electron mobility, the charge recombination, and the morphology explain why PTB7‐Th:SubPcCl_6_‐X BHJ solar cells show low FF.

**Figure 5:**
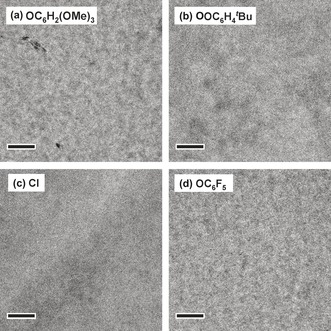
Bright‐field TEM images of the PTB7‐Th:SubPcCl_6_‐X blend films deposited with the same methods as those for OSC fabrication. Image size: 1.5×1.5 μm^2^; scale bar: 200 nm.

In conclusion, hexachlorosubphthalocyanines bearing different axial substituents have been synthesized and used as electron acceptors in BHJ polymer solar cells. A PCE up to 4.0 % was achieved, which is the highest reported value for solution‐processed SubPc‐based solar cells. Both the polymer donor and the SubPc acceptor contribute significantly to the photocurrent, indicating promising acceptor properties of subphthalocyanines. The main limitation of these SubPc‐based solar cells is their low fill factor, which is a collective result of low electron mobility, serious bimolecular recombination, and suboptimal BHJ morphology. Further research involving subphthalocyanines and structurally related systems such as subnaphthalocyanines should therefore focus on improving electron mobility, avoiding geminate recombination, and controlling microstructure in solid state through rational molecular design.

## Supporting information

As a service to our authors and readers, this journal provides supporting information supplied by the authors. Such materials are peer reviewed and may be re‐organized for online delivery, but are not copy‐edited or typeset. Technical support issues arising from supporting information (other than missing files) should be addressed to the authors.

Supplementary
