# Supplementary material for: The Role of the Axial Substituent in Subphthalocyanine Acceptors for Bulk‐Heterojunction Solar Cells
Source: Angew Chem Int Ed Engl. 2016 Nov 28;56(1):148–52. doi: 10.1002/anie.201608644 (PMC6680215; doi:10.1002/anie.201608644)
Supplement: Supplementary file 1 — Supplementary [file ANIE-56-148-s001.pdf]

Angewandte Chemie
Eine Zeitschrift der Gesellschaft Deutscher Chemiker

Supporting Information

The Role of the Axial Substituent in Subphthalocyanine Acceptors for Bulk-Heterojunction Solar Cells

Chunhui Duan[+], Germán Zango[+], Miguel García Iglesias, Fallon J. M. Colberts, Martijn M. Wienk, M. Victoria Martínez-Díaz, René A. J. Janssen,* and Tomás Torres**

anie_201608644_sm_miscellaneous_information.pdf

1. Synthesis

All chemicals were purchased from Sigma-Aldrich Co., TCI Europe N.V. and Alfa Aesar and used without further purification. Solvents were purchased from Carlo Erba Reagents and Scharlab. 4,5-dichlorophthalonitrile[1] was prepared using a described procedure. Column chromatography was carried out on silica gel Merck-60 and TLC was carried out on aluminium sheets percolated with silica gel 60 F254 (Merck).

SubPcCl$_6$-Cl: this compound was synthesized according to the procedure previously described.[2] mp> 250 °C; ^{1}H NMR (300 MHz, $CDCl_3$, δ): 8.93 ppm (s, 6H); ^{13}C NMR (75.5 MHz, $CDCl_3$, δ): 150.5, 135.1, 129.5, 123.7 ppm; UV–vis (Toluene): λ_{max} (log ε) = 572 (4.6), 552 (sh), 528 (4.1), 310 nm (4.3); MALDI-TOF MS m/z (%): 637.9 (100) [M^+]; HRMS (MALDI-TOF) m/z : $[M]^+$ calcd for $C_{24}H_6N_6BCl_7$, 633.8566; found, 633.8532; FT-IR (KBr), υ: 2920, 2851, 1606, 1539, 1456, 1419, 1373, 1278, 1222, 1095, 1042, 976, 882, 819 cm^{-1}.

SubPcCl$_6$-OPhF$_5$: in a 25 mL two-necked round-bottomed flask, equipped with a condenser, magnetic stirrer and rubber seal, pentafluorophenol (550 mg, 3 mmol), SubPcCl$_6$-Cl (320 mg, 0.50 mmol) and DBU (75 µL, 0.50 mmol) were refluxed in toluene (3 mL) for 12 h. After cooling down to room temperature, the excess of phenol was removed by washing the crude with a 3:1 MeOH/water solution. The resulting dark solid was subjected to column chromatography on silica gel using toluene/heptane (4:1) as an eluent. By washing with heptane, SubPcCl$_6$-OPhF$_5$ was further purified, obtaining a purple solid (264 mg, 67%). Physical characteristics of SubPcCl$_6$-OPhF$_5$ adequately match previously reported characterization.[3] mp> 250 °C; ^{1}H NMR (300 MHz, $CDCl_3$, δ): 8.90 ppm (s, 6H); ^{13}C NMR (75.5 MHz, $CDCl_3$, δ): 150.2, 135.7, 130.8, 130.3, 129.9, 129.2, 128.7, 124.2 ppm; UV–vis

(Toluene): λ_{max} (log ε) = 574 (4.6), 556 (sh), 532 (4.1), 314 nm (4.3); MALDI-TOF MS m/z (%): 783.9 (100) [M^+], 600.9 (90) [M - axial group]$^+$; HRMS (MALDI-TOF) m/z : [M]$^+$ calcd for $C_{30}H_6BCl_6F_5N_6O$, 783.8720; found, 783.8663; FT-IR (KBr), υ: 2923, 2853, 1611, 1510, 1458, 1422, 1377, 1280, 1223, 1183, 1096, 1047, 992, 887, 822, 766, 710 cm^{-1}.

SubPcCl$_6$-OPh^{t}Bu: in a 25 mL two-necked round-bottomed flask, equipped with a condenser, magnetic stirrer and rubber seal, 4-*tert*-butylphenol (450 mg, 3 mmol), SubPcCl$_6$-Cl (320 mg, 0.50 mmol) and DBU (75 µL, 0.50 mmol) were refluxed in toluene (3 mL) for 8 h. After cooling down to room temperature, the excess of phenol was removed by washing the crude with a 3:1 MeOH/water solution. The resulting dark solid was subjected to column chromatography on silica gel using toluene/heptane (3:1) as an eluent. By washing with heptane, SubPcCl$_6$-OPh^{t}Bu was further purified, obtaining a purple solid (320 mg, 85%). mp> 250 °C; ^{1}H NMR (300 MHz, $CDCl_3$, δ): 8.84 (s, 6H), 6.80 (d, J_o = 8.6 Hz, 2H), 5.31 (d, J_o = 8.6 Hz, 2H), 1.11 ppm (s, 9H); ^{13}C NMR (75.5 MHz, $CDCl_3$, δ): 150.5, 149.5, 135.2, 132.5, 129.9, 126.1, 124.0, 118.0, 34.0, 31.4 ppm; UV–vis (Toluene): λ_{max} (log ε) = 571 (4.6), 522 (sh), 308 nm (4.3); MALDI-TOF MS m/z (%): 750.0 (100) [M^+], 600.9 (20) [M - axial group]$^+$; HRMS (MALDI-TOF) m/z : [M]$^+$ calcd for $C_{34}H_{19}BCl_6N_6O$, 749.9819; found, 749.9821; FT-IR (KBr), υ: 2960, 2924, 2865, 1608, 1513, 1458, 1419, 1256, 1219, 1186, 1096, 1066, 885, 822, 709 cm^{-1}.

SubPcCl$_6$-OPh(OMe)$_3$: in a 25 mL two-necked round-bottomed flask, equipped with a condenser, magnetic stirrer and rubber seal, 3,4,5-trimethoxyphenol (550 mg, 3 mmol) and SubPcCl$_6$-Cl (320 mg, 0.50 mmol) were refluxed in toluene (3 mL) for 6 h. After cooling down to room temperature, the excess of phenol was removed by washing the crude with a

3:1 MeOH/water solution. The resulting dark solid was subjected to column chromatography on silica gel using $CHCl_3$/MeOH (500:1) as an eluent. By washing with heptane, $SubPcCl_6$-$OPh(OMe)_3$ was further purified, obtaining a purple solid (178 mg, 45%). mp> 250 °C; ^1H NMR (300 MHz, $CDCl_3$, δ): 8.61 (s, 6H), 4.55 (s, 2H), 3.48 (s, 3H), 3.44 ppm (s, 6H); ^{13}C NMR (75.5 MHz, $CDCl_3$, δ): 152.9, 150.2, 148.3, 134.9, 132.8, 129.5, 123.6, 95.9, 60.6, 55.8 ppm; UV–vis (Toluene): λ_{max} (log ε) = 572 (4.6), 554 (sh), 530 (4.1), 312 nm (4.3); MALDI-TOF MS m/z (%): 784.0 (100) [M^+], 618 (40) [M - axial group + OH]$^+$; HRMS (MALDI-TOF) m/z : [M]$^+$ calcd for $C_{33}H_{17}BCl_6N_6O_4$, 783.9509; found, 783.9509; FT-IR (KBr), υ: 3081, 3002, 2934, 2835, 1735, 1594, 1543, 1504, 1461, 1422, 1379, 1341, 1281, 1221, 1196, 1127, 1095, 1068, 1008, 886, 822, 762, 708 cm^{-1}.

2. Measurements and characterization

^1H NMR and ^{13}C NMR spectra were obtained using a Bruker Avance 300 spectrometer or a Bruker DRX-500 spectrometer. Matrix-assisted laser desorption/ionization time of flight (MALDI-TOF) MS and high-resolution mass spectrometry (HRMS) spectra were recorded with a Bruker Reflex III spectrometer. Infrared (IR) spectra were recorded on a Bruker Vector 22 spectrophotometer, employing in all cases solid samples (KBr pressed disks). UV-visible absorption spectra were recorded with a Jasco V-660 instrument. Fluorescence studies were carried out with a JASCO-V8600 fluorometer. Fluorescence quantum yields were determined using $SubPcH_{12}$-Cl (φ_F = 0.73) in THF as standard.[4] Electrochemical measurements were performed with an Autolab PGStat 30 system using a three electrode

configuration system. The measurements were carried out using THF solutions containing 0.1 M tetrabutylammonium hexafluorophosphate ($TBAPF_6$). A glassy carbon electrode (3 mm diameter) was used as the working electrode, and a platinum wire and an $Ag/AgNO_3$ (in CH_3CN) electrode were employed as the counter and the reference electrodes, respectively. Ferrocene (Fc) was added as an internal reference and all the potentials were given relative to the Fc/Fc^+ couple. The scan rate was 100 mV/s. LUMO energies were estimated from the redox potentials derived from CV data, and were calculated using the approximation:[5]

$$E_{LUMO} = -5.1 - E^1_{1/2,red}\ (vs.\ Fc/Fc^+)\ (eV)$$

HOMO energies values were obtained from LUMO values and optical band gap E^g_{opt} values, which were estimated from the intersection of the normalized absorption and emission spectra. Transmission electron microscopy (TEM) was performed on a Tecnai G^2 Sphera transmission electron microscope (FEI) operated at 200 kV. Space-charge-limited-current electron mobility was acquired through the electron-only devices with a configuration of ITO/ZnO (40 nm)/PTB7-Th:$SubPcCl_6$-X/LiF (1 nm)/Al (100 nm). The thickness of polymer:[70]PCBM blend films is about 300 nm. The dark current densities of PTB7-Th:$SubPcCl_6$-X blends were measured by applying a voltage between 0 and 4 V using a computer-controlled Keithley 2400 source meter in N_2atmosphere. These data were analysed according to the Mott-Gurney laws that includes a Poole-Frenkel-type dependence of mobility on the electric field, given by $J = \frac{9}{8}\varepsilon_r \varepsilon_0 \mu_0 \frac{V^2}{d^3} \exp(0.89\gamma\sqrt{\frac{V}{d}})$, where ε_0 is the permittivity of free space, ε_r is the dielectric constant of the polymer which is assumed to be around 3 for the conjugated polymers, μ_0 is the zero-field mobility, V is the voltage drop

across the device, d is the film thickness of active layer, and γ is a parameter that describes the strength of the field-dependence effect. The applied voltage is used without correcting from series resistance or built-in voltage, which offers the best fitting of the experimental data following the protocol reported in literature.[6] The electron mobilities are extracted with the fit parameters at an electric field (E) of 1×10^5 V cm^{-1} (corresponding to an applied voltage of 3 V across the bulk of a 300 nm device) by the Murgatroyd equation $\mu = \mu_0 \exp(\gamma\sqrt{E})$.

3. Fabrication and characterization of solar cells

Photovoltaic devices were made with an inverted architecture (glass/ITO/ZnO/ PTB7-Th:SubPcCl_6-X/MoO_x/Ag. Patterned ITO substrates (14 Ω per square) (Naranjo Substrates) were cleaned by sonication in acetone, detergent, deionized water, and isopropanol, followed by UV-ozone treatment. ZnO layers were deposited by spin coating a zinc acetate dehydrate (100 mg) precursor solution (28.3 μL ethanolamine in 0.937 mL 2-methoxyethanol) at 4000 rpm for 60 seconds, followed by annealing at 200 °C for 10–15 min in air, giving layers of 40 nm. The PTB7-Th:SubPcCl_6-X photoactive layers were deposited by spin coating in air from the solutions containing PTB7-Th and corresponding SubPcCl_6-X at room temperature. Active layer thickness were about 75 nm. Unless indicated otherwise, Thermal annealing were performed in glovebox at indicated temperature for 5 min. MoO_x (10 nm) and Ag (100 nm) were deposited by vacuum evaporation at ~3×10^{-7} mbar as the back electrode. The active area of the cells was 0.09 or 0.16 cm^2, which provided similar

results. Current density–voltage ($J-V$) curves were measured under simulated solar light (100 mW cm^{-2}) from a tungsten–halogen lamp filtered by a Hoya LB100 daylight using a Keithley 2400 source meter. No mismatch correction was done. All measurements were conducted in nitrogen-filled glove box. The accurate short-circuit current density (J_{sc}) was determined from the EQE by convolution with the AM1.5G solar spectrum. External quantum efficiency (EQE) measurements were performed in a homebuilt set-up, with the devices kept in a nitrogen filled box with a quartz window and illuminated through an aperture of 2 mm. Mechanically modulated (Stanford Research, SR 540) monochromatic (Oriel, Cornerstone 130) light from a 50 W tungsten halogen lamp (Osram 64610) was used as probe light, in combination with continuous bias light from a solid state laser (B&W Tek Inc. 532 nm, 30 mW). The intensity of the bias laser light was adjusted using a variable-neutral density filter. The response was recorded as the voltage over a 50 Ω resistance, using a lock-in amplifier (Stanford Research Systems SR 830). For all devices, the measurements were carried out under representative illumination intensity (AM1.5G equivalent, provided by the 532 nm laser).

4. Additional Figures and Tables

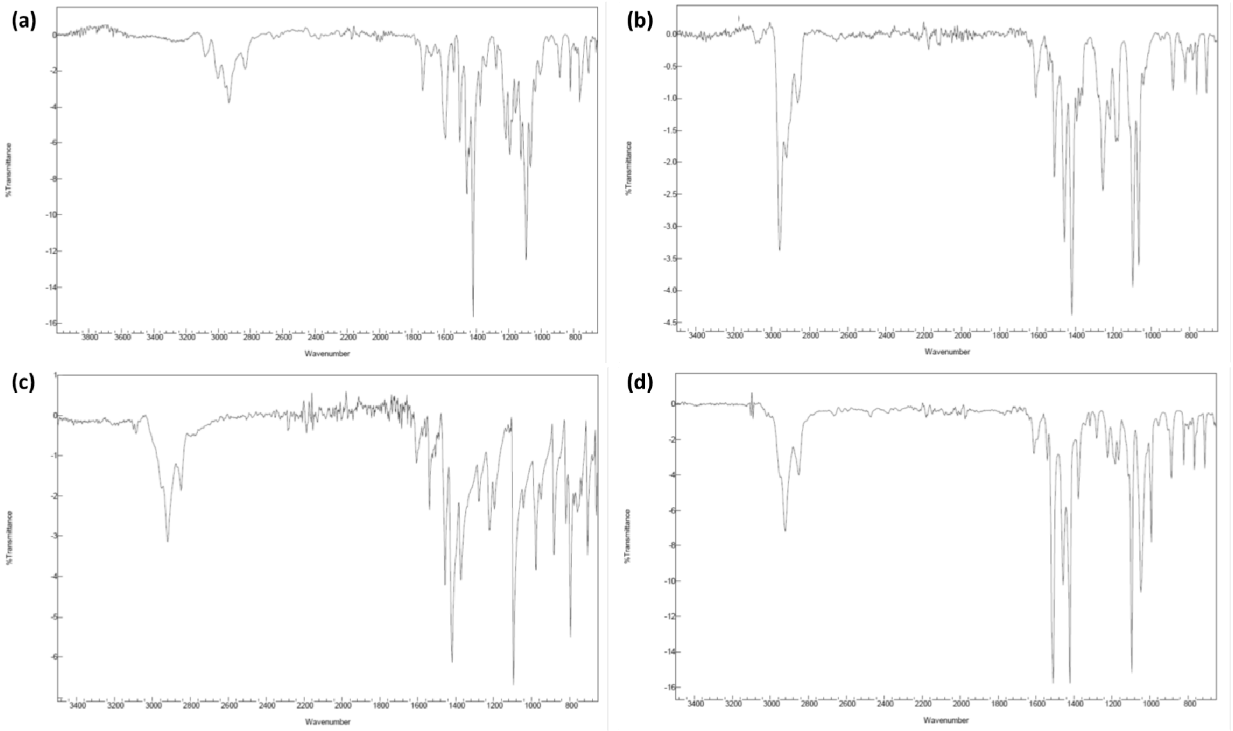

Figure S1. IR spectra of (a) SubPcCl$_6$-OPh(OMe)$_3$, (b) SubPcCl$_6$-OPh^{t}Bu, (c) SubPcCl$_6$-Cl, and (d) SubPcCl$_6$-OPhF$_5$.

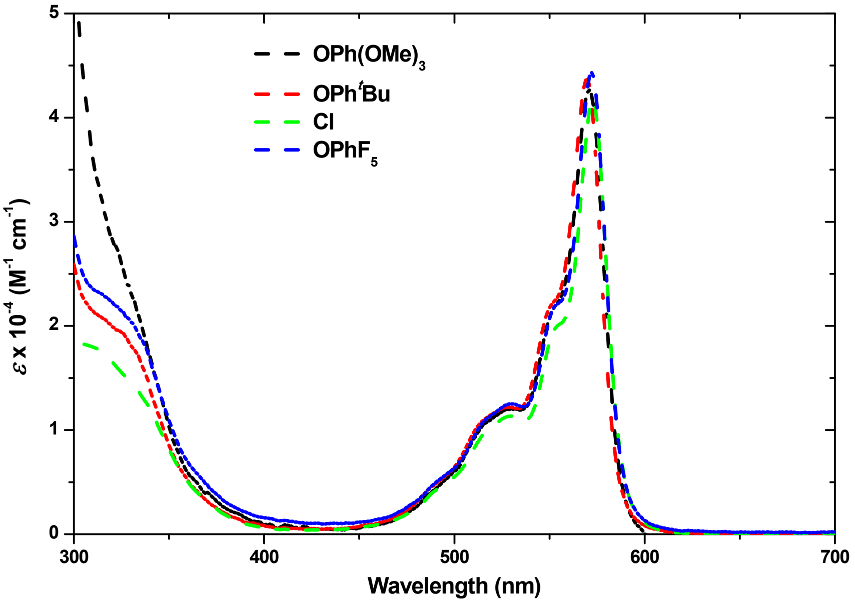

Figure S2. UV-vis absorption spectra of SubPcCl$_6$-X in toluene.

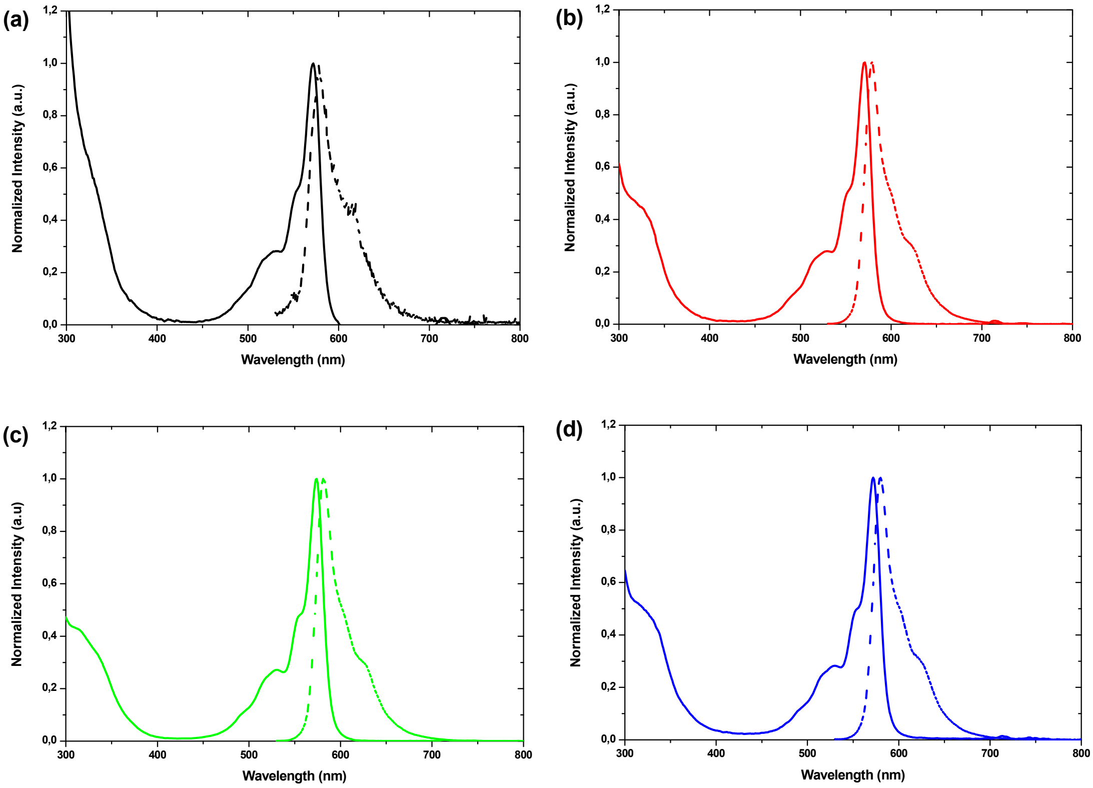

Figure S3. Normalized UV-vis absorption spectra (solid line) and fluorescence emission spectra (dotted line, exc. wavelength = 520 nm) (dark red) of (a) $SubPcCl_6$-$OPh(OMe)_3$, (b) $SubPcCl_6$-OPh^tBu, (c) $SubPcCl_6$-Cl, and (d) $SubPcCl_6$-$OPhF_5$ in toluene.

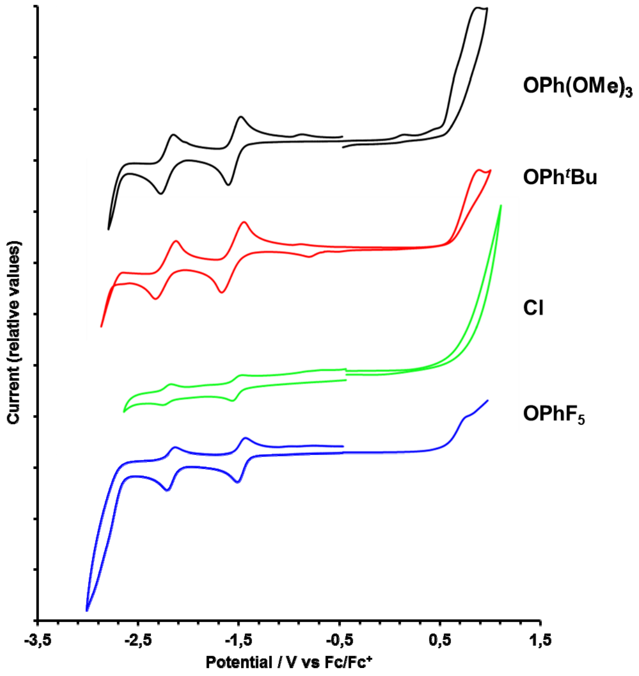

Figure S4. Cyclic voltammograms (OSW voltammogram in the anodic window of $SubPcCl_6$-$OPhF_5$) of compounds $SubPcCl_6$-X (referred to Fc/Fc^+) in THF.

Table S1. Performance parameters of the PTB7-Th:SubPcCl$_6$-X solar cells in device structure of ITO/ZnO (40 nm)/PTB7-Th:SubPcCl$_6$-X/MoO$_x$ (10 nm)/Ag (100 nm) under different conditions.

SubPcCl$_6$-X	D:A	Solvent	Annealing	J_{sc} (mA/cm^2)	V_{oc} (V)	FF	P_{max} (mW cm^{-2})	PCE (%)
OPh(OMe)$_3$	1:1.5	CB (2% CN)	90 °C, 5 min	4.19	0.664	0.346	0.94	0.74
		CB (2% DIO)	-	**4.52**	**0.735**	**0.404**	**1.34**	**1.07**
			90 °C, 5 min	4.14	0.712	0.403	1.19	
OPh^{t}Bu	1:1.5	CB (2% CN)	90 °C, 5 min	**6.56**	**0.807**	**0.406**	**2.15**	**1.75**
		CB (2% DIO)	-	4.22	0.728	0.384	1.18	
			90 °C, 5 min	4.35	0.746	0.397	1.29	
Cl	1.5:1	CB (3% DIO)	-	5.65	0.781	0.458	2.02	
	1:1	CB	-	9.37	0.727	0.412	2.81	
		CB (3% DIO)	-	6.20	0.787	0.462	2.25	
		CB (1% CN)	-	8.10	0.714	0.383	2.21	
		CB (2% CN)	-	9.59	0.724	0.418	2.90	
			120 °C, 5 min	10.40	0.759	0.437	3.43	3.06
		CB (3% CN)	-	7.84	0.663	0.403	2.09	
		CB (4% CN)	-	6.05	0.691	0.364	1.52	
		CB (2% DPE)	-	7.91	0.693	0.398	2.18	
		CB (2% pyridine)	-	5.69	0.776	0.381	1.68	
			120 °C, 5 min	5.82	0.779	0.353	1.60	
	1:1.5	CB	-	11.90	0.777	0.433	4.02	3.41
		CB (3% DIO)	-	6.76	0.783	0.486	2.57	2.41
		CB (2% CN)	-	9.80	0.736	0.441	3.18	2.89
			60 °C, 5 min	11.10	0.773	0.485	4.16	3.89
			90 °C, 5 min	**11.10**	**0.770**	**0.480**	**4.20**	**3.95**
			120 °C, 5 min	11.00	0.765	0.462	3.88	3.62
		CF (3% CN)	-	11.20	0.770	0.458	3.95	3.38
			90 °C, 5 min	11.70	0.771	0.474	4.27	3.76
	1:2	CB (3% DIO)	-	6.24	0.798	0.481	2.39	
OPhF$_5$	1:1.5	CB (2% CN)	90 °C, 5 min	**2.49**	**0.503**	**0.454**	**0.57**	**0.48**
		CB (2% DIO)	-	2.43	0.513	0.432	0.54	
			90 °C, 5 min	2.37	0.518	0.445	0.55	

Table S2. Device statistics of PTB7-Th:SubPcCl6-X solar cells.[a]

SubPcCl$_6$-X	J_{sc} (mA cm^{-2})	J_{sc}(EQE)[b] (mA cm^{-2})	V_{oc} (V)	FF (-)	P_{max} (mW cm^{-2})	PCE[c] (%)
OPh(OMe)$_3$	4.5<br>4.5 ± 0.1	3.6	0.74<br>0.73 ± 0.01	0.40<br>0.40 ± 0.01	1.3<br>1.3 ± 0.1	1.1
OPh^{t}Bu	6.6<br>6.5 ± 0.1	5.3	0.81<br>0.81 ± 0.01	0.41<br>0.41 ± 0.01	2.2<br>2.1 ± 0.1	1.8
Cl	11.4<br>11.2 ± 0.2	10.7	0.77<br>0.77 ± 0.01	0.48<br>0.48 ± 0.01	4.2<br>4.1 ± 0.1	4.0
OPhF$_5$	2.5<br>2.4 ± 0.1	2.1	0.50<br>0.50 ± 0.01	0.45<br>0.45 ± 0.01	0.6<br>0.6 ± 0.1	0.5

[a]Measured with white light (100 mW cm^{-2}) in a ITO/ZnO (40 nm)/PTB7-Th:SubPcCl$_6$-X/MoO$_x$ (10 nm)/Ag (100 nm) device configuration. Both maximum and device statistics from four devices are given. [b]Determined by integrating the EQE with the AM1.5G solar spectrum. [c]Calculated using J_{sc} (EQE).

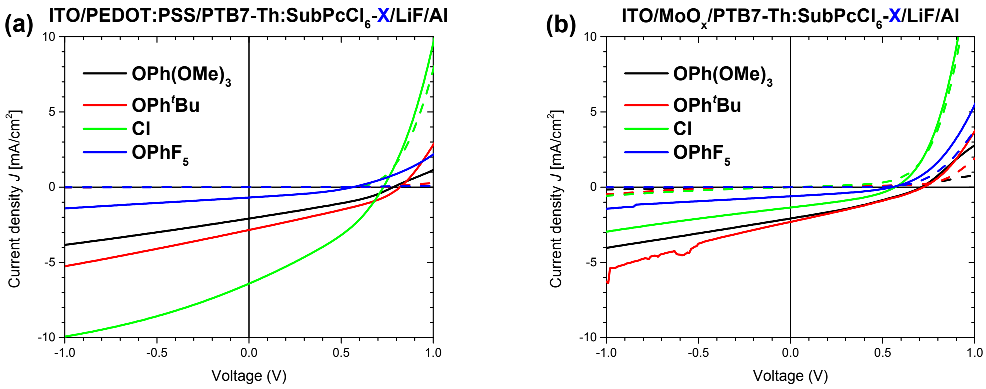

Figure S5. Current density–voltage (*J–V*) characteristics of the PTB7-Th:SubPcCl$_6$-X solar cells in conventional device structure with different hole-collecting contacts in dark and under AM1.5G illumination (100 mW cm^{-2}): (a) PEDOT:PSS; (b) MoO$_x$.

Table S3. Performance parameters of the PTB7-Th:$SubPcCl_6$-X solar cells with optimized active layer processing conditions in device structure of ITO/HCL/PTB7-Th:$SubPcCl_6$-X (1:1.5)/LiF (1 nm)/Al (100 nm) under AM1.5G illumination (100 mW cm^{-2}).

$SubPcCl_6$-X	HCL	J_{sc} (mA cm^{-2})	V_{oc} (V)	FF	PCE (%)
$OPh(OMe)_3$	PEDOT:PSS	2.1	0.78	0.33	0.5
	MoO_x	2.1	0.71	0.32	0.5
OPh^{t}Bu	PEDOT:PSS	2.9	0.82	0.32	0.8
	MoO_x	2.3	0.72	0.29	0.5
Cl	PEDOT:PSS	6.4	0.73	0.35	1.6
	MoO_x	1.4	0.57	0.36	0.3
$OPhF_5$	PEDOT:PSS	0.7	0.56	0.35	0.1
	MoO_x	0.6	0.55	0.35	0.1

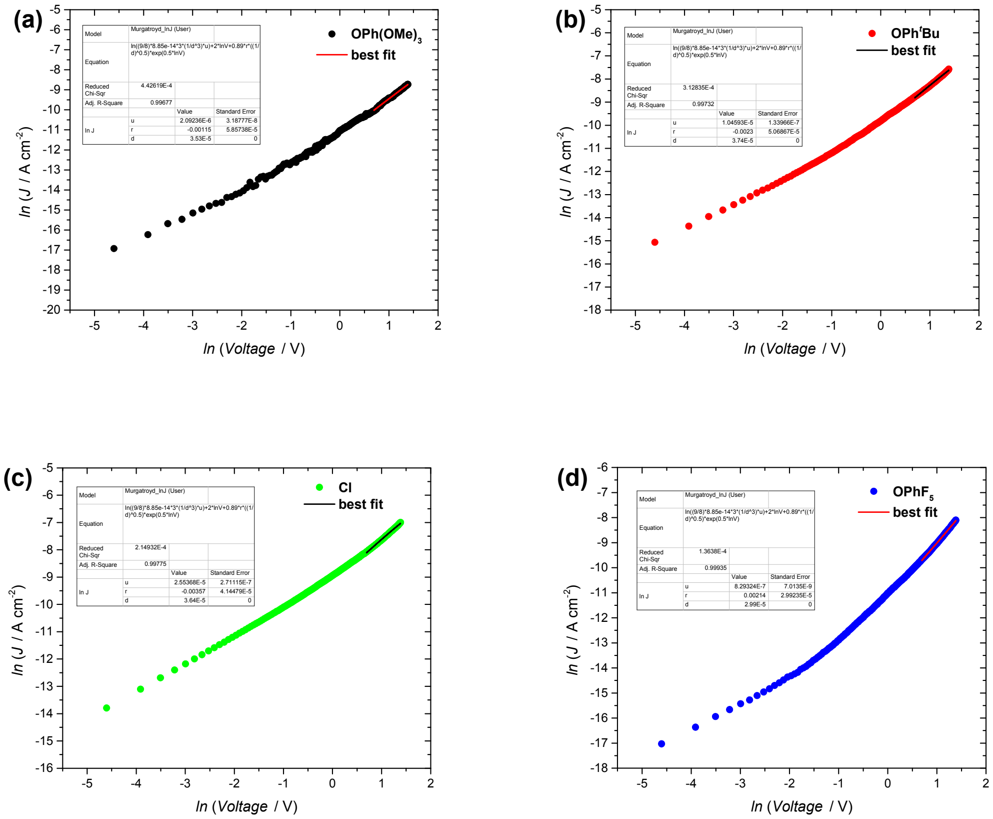

Figure S6. Current density versus voltage characteristics of electron-only devices with a configuration of ITO/ZnO (40 nm)/PTB7-Th:$SubPcCl_6$-X/LiF (1 nm)/Al (100 nm) plotted in the format of ln$J$ ~ ln$V$: (a) $OPh(OMe)_3$, (b) OPh^{t}Bu, (c) Cl, and (d) $OPhF_5$.

Table S4. Summary of the derived fitting data for the electron-only devices of ITO/ZnO (40 nm)/PTB7-Th:SubPcCl$_6$-X/LiF (1 nm)/Al (100 nm) based on Mott-Gurneys law with field-dependent mobility.

SubPcCl$_6$-X	Zero-field mobility μ_0 (cm^2 V^{-1} s^{-1})	Field-dependence factor γ (cm$^{1/2}$ V$^{-1/2}$)	μ_h at $E = 1 \times 10^5$ V cm^{-1} (cm^2 V^{-1} s^{-1})
OPh(OMe)$_3$	2.1×10^{-6}	-1.2×10^{-3}	1.5×10^{-6}
OPh^{t}Bu	1.1×10^{-5}	-2.3×10^{-3}	5.1×10^{-6}
Cl	2.6×10^{-5}	-3.6×10^{-3}	8.3×10^{-6}
OPhF$_5$	8.3×10^{-7}	2.1×10^{-4}	1.6×10^{-6}

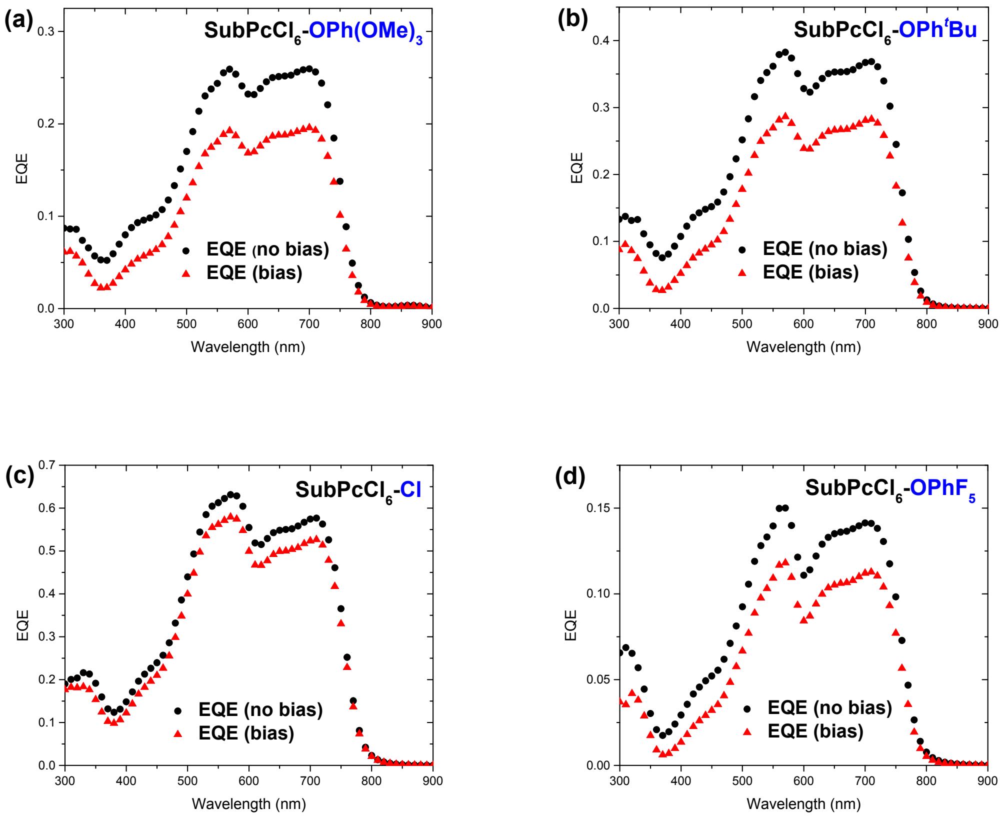

Figure S7. EQE spectra measured with and without light bias of the ITO/ZnO (40 nm)/PTB7-Th:SubPcCl$_6$-X/MoO$_x$ (10 nm)/Ag (100 nm) solar cells: (a) OPh(OMe)$_3$, (b) OPh^{t}Bu, (c) Cl, and (d) OPhF$_5$.

References

1 D. Wöhrle, M. Eskes, K. Shigehara, A. Yamada, *Synthesis* **1993**, 2, 194.

2 B. Verreet, K. Cnops, D. Cheyns, P. Heremans, A. Stesmans, G. Zango, C. G.

Claessens, T. Torres, B. P. Rand, *Adv. Energy Mater.* **2014**, 4, 1301413.

3 G. E. Morse, M. G. Helander, J. Stanwick, J. M. Sauks, A. S. Paton, Z. H. Lu, T. P. Bender, *J. Phys. Chem. C* **2011**, 115, 11709.

4 M. V. Fulford, D. Jaidka, A. S. Paton, G. E. Morse, E. R. L. Brisson, A. J. Lough, T. P. Bender, *J. Chem. Eng. Data* **2012**, 57, 2756.

5 C. M. Cardona, W. Li, A. E. Kaifer, D. Stockdale, G. C. Bazan, *Adv. Mater.* **2011**, 23, 2367.

6 J. C. Blakesley, F. A. Castro, W. Kylberg, G. F. A. Dibb, C. Arantes, R. Valaski, M. Cremona, J. S. Kim, J.-S. Kim, *Org. Electron.* **2014**, 15, 1263.
